# Real-World Emulation of Landmark Lung Cancer Trials: A Registry-Based Reconstruction of KN189, KN407, IMpower133, and PACIFIC

**DOI:** 10.3390/cancers18111754

**Published:** 2026-05-27

**Authors:** Irina Surovtsova, Wilfried E. E. Eberhardt, Dorothea E. Meschke, Philipp Morakis

**Affiliations:** 1 Clinical State Registry Baden-Württemberg GmbH, Baden-Württemberg Cancer Registry (BWCR), 70191 Stuttgart, Germany; surovtsova@klr-krbw.de; 2 Department of Medical Oncology, West German Cancer Center, University Hospital Essen, University Duisburg-Essen, 45147 Essen, Germany; wilfried.eberhardt@uni-duisburg-essen.de (W.E.E.E.); dorothea.e.meschke@med.uni-giessen.de (D.E.M.); 3 Quality Conferences Office, Clinical State Registry Baden-Württemberg GmbH, Baden-Württemberg Cancer Registry (BWCR), 70191 Stuttgart, Germany

**Keywords:** lung cancer, target trial emulation, real-world data

## Abstract

**Background:** Checkpoint inhibitors (CPIs) improve the survival in advanced NSCLC and ES-SCLC, but reproducibility of randomized trial results in routine practice remains unclear. Target trial emulation enables approximation of randomized trial designs using observational data while reducing common biases. **Methods:** Using the Baden-Württemberg Cancer Registry, we emulated four pivotal trials: KEYNOTE-189 (eKN189), KEYNOTE-407 (eKN407), IMpower133 (eIMP133), and PACIFIC (ePACIFIC). The eligibility criteria, treatment strategies, and outcomes were aligned with the original trials as closely as possible within registry constraints. Time-zero was defined as the treatment initiation for systemic therapy cohorts (eKN189, eKN407, eIMP133) and as completion of chemoradiotherapy for ePACIFIC. The treatment groups were balanced using propensity-score-based inverse probability weighting. The outcomes included the overall survival (OS) and the objective response rate (ORR). **Results:** A total of 4015 patients were included (eKN189: 1762; eKN407: 467; eIMP133: 1190; ePACIFIC: 595). After weighting, the baseline covariates were well balanced. ICI-based regimens were associated with an improved survival: eKN189: HR—0.59 (95% CI: 0.52–0.66), 5-year OS—22.7% vs. 4.7%; eKN407: HR—0.69 (95% CI: 0.54–0.89), 5-year OS—11.8% vs. 6.6%; eIMP133: HR—0.7 (95% CI: 0.62–0.79), 5-year OS—12.8% vs. 5.3%; ePACIFIC: HR—0.51 (95% CI: 0.39–0.66), 5-year OS—47.3% vs. 23.4%. The OS curves demonstrated long-term plateaus across CPI-containing arms. The ORRs were consistently higher with CPI-containing regimens (eKN189: 41.6% vs. 29.6%; eKN407: 44.8% vs. 31.5%; eIMP133: 51.7% vs. 43.1%; ePACIFIC: 56.6% vs. 47.1%). **Conclusions:** This registry-based target trial emulation reproduced the survival patterns observed in four landmark lung cancer RCTs within trial-like real-world populations, supporting the external validity of pivotal immunotherapy trials while reinforcing the role of high-quality registries as a powerful complement to randomized trials in oncology.

## Simple Summary

In this study, we examined whether the benefits of modern immunotherapy for lung cancer observed in clinical trials can also be confirmed in everyday medical practice. Because clinical trials are highly controlled and may not fully reflect real-world patient populations, we used a clean target trial emulation approach based on a large population-based cancer registry from Baden-Württemberg. This method allows us to closely recreate the design of major randomized trials while minimizing common biases in observational data. Our aim was to assess whether immune checkpoint inhibitor therapies provide similar survival advantages outside of trial settings. We found that the emulated trial results closely matched the original studies, showing consistent improvements in the survival and treatment response. These findings support the validity of rigorous trial emulation in real-world oncology research and highlight its value for generating reliable evidence that complements randomized controlled trials.

## 1. Introduction

Randomized controlled trials (RCTs) have transformed the therapeutic landscape of lung cancer. Landmark trials such as KEYNOTE-189 [1,2,3,4,5], KEYNOTE-407 [6,7,8], IMpower133 [9,10,11] and PACIFIC [12,13,14] established immune checkpoint inhibitors (ICIs) as a standard of care across various subtypes and stages of lung cancer, including metastatic non-small cell lung cancer (mNSCLC), extensive-stage small cell lung cancer (ES-SCLC), and unresectable stage III NSCLC. These trials demonstrated substantial improvements in survival and led to a paradigm shift in first-line and consolidation treatment of advanced disease.

Despite their strong internal validity, the generalizability of RCT findings to routine oncology practice remains an important clinical question. Trial populations are selected through strict eligibility criteria and treated under highly protocolized conditions, whereas patients in routine oncology practice often present with greater clinical heterogeneity, comorbidities and variation in treatment delivery [15,16,17,18,19,20]. Consequently, whether the magnitude of benefit observed in pivotal immunotherapy trials can be reproduced in routine care remains clinically relevant.

Real-world data (RWD), particularly from population-based cancer registries, provide an opportunity to evaluate treatment outcomes under routine care conditions and complement the evidence generated by RCTs [15,16,17,18,19,20]. However, conventional observational comparisons may yield biased estimates if they fail to adequately replicate the design, timing and treatment assignment structure of the trials they aim to assess. To strengthen causal interpretation, the target emulation framework explicitly specifies the protocol of a hypothetical randomized trial underlying the observational analysis, thereby reducing common biases such as an immortal time bias, a selection bias or an inappropriate adjustment for post-baseline variables [21,22,23]. This framework has emerged as a key methodological approach for improving the causal inference from real-world data when randomized evidence is unavailable or when assessing the transportability of trial findings to broader populations [24].

Guided by this framework, we emulated four landmark lung cancer RCTs using data from the Baden-Württemberg Cancer Registry (BWCR), a population-based registry covering more than 11 million inhabitants of Germany. For each emulation, the eligibility criteria, treatment strategies, time-zero, follow-up, and endpoints were aligned as closely as possible with the corresponding trial protocol within the constraints of the registry data. To approximate randomization, inverse probability of the treatment weighting (IPTW) based on propensity scores was applied according to established best practices for causal inference in observational studies [25,26,27].

To our knowledge, this is the first large-scale study to emulate four pivotal lung cancer immunotherapy trials within a unified methodological framework using a single population-based registry.

By rigorously reconstructing landmark trials in routine care populations, this study evaluates the external validity and transportability of randomized evidence to clinical practice. Concordant findings would strengthen the confidence that the survival benefits observed in KEYNOTE-189, KEYNOTE-407, IMpower133, and PACIFIC are achievable beyond controlled trial environments, whereas clinically meaningful deviations may identify patient groups or treatment settings in which the real-world effectiveness differs from the trial efficacy.

## 2. Materials and Methods

### 2.1. Study Design and Target Trial Emulation

We conducted a retrospective, population-based cancer registry study designed to emulate four landmark RCTs (KEYNOTE-189, KEYNOTE-407, IMpower133, and PACIFIC), using data from the BWCR. Each analysis followed the target trial emulation framework described by Hernán and Robins [21,22,23], including explicit specification of the eligibility criteria, treatment strategies, assignment procedures, time-zero, follow-up, and outcome definitions for each emulated trial. A detailed comparison between the original RCT protocols and the corresponding registry-based emulations, including the eligibility criteria, treatment strategies, time-zero definitions, endpoints, and major deviations, is provided in Supplementary Table S1.

### 2.2. Data Source

Patient-level data were obtained from the Clinical Cancer Registry of the Federal State of Baden-Württemberg Germany, which covers a population of more than 11 million (as of 2019). The registry collects standardized information on the diagnosis, treatment, and longitudinal follow-up outcomes for all cancer patients in Baden-Württemberg, based on mandatory reporting from all relevant healthcare providers across the state. Data collection follows legally mandated, standardized, and anonymized procedures. The registry operates under strict quality assurance protocols, including routine completeness checks and plausibility validations to ensure data accuracy and reliability.

### 2.3. Eligibility Criteria and Study Population

The study population included adult patients (aged ≥ 18 years) residing in Baden-Württemberg who were diagnosed between 2016 and 2024 with advanced or metastatic non-small cell lung cancer (mNSCLC) or extensive-stage small cell lung cancer (ES-SCLC). To approximate the eligibility criteria of the target trials, inclusion was restricted to patients with an ECOG performance status of 0 or 1 at baseline. The eligibility criteria were operationalized using variables available in the registry, and therefore represent clinically meaningful approximations rather than exact replications of the original protocols. Trial-specific cohorts were defined as follows:

- KEYNOTE-189 emulation (eKN189): Patients with non-squamous mNSCLC treated with cisplatin or carboplatin plus pemetrexed, with or without pembrolizumab.
- KEYNOTE-407 emulation (eKN407): Patients with squamous mNSCLC treated with carboplatin plus paclitaxel or nab-paclitaxel, with or without pembrolizumab.
- IMpower133 emulation (eIMP133): Patients with ES-SCLC treated with carboplatin plus etoposide, with or without atezolizumab.
- PACIFIC emulation (ePACIFIC): Patients with stage III NSCLC treated with concurrent chemoradiotherapy, with or without durvalumab consolidation. Unresectability was defined by absence of tumor-specific surgery (based on OPS codes).

**Figure 1:**
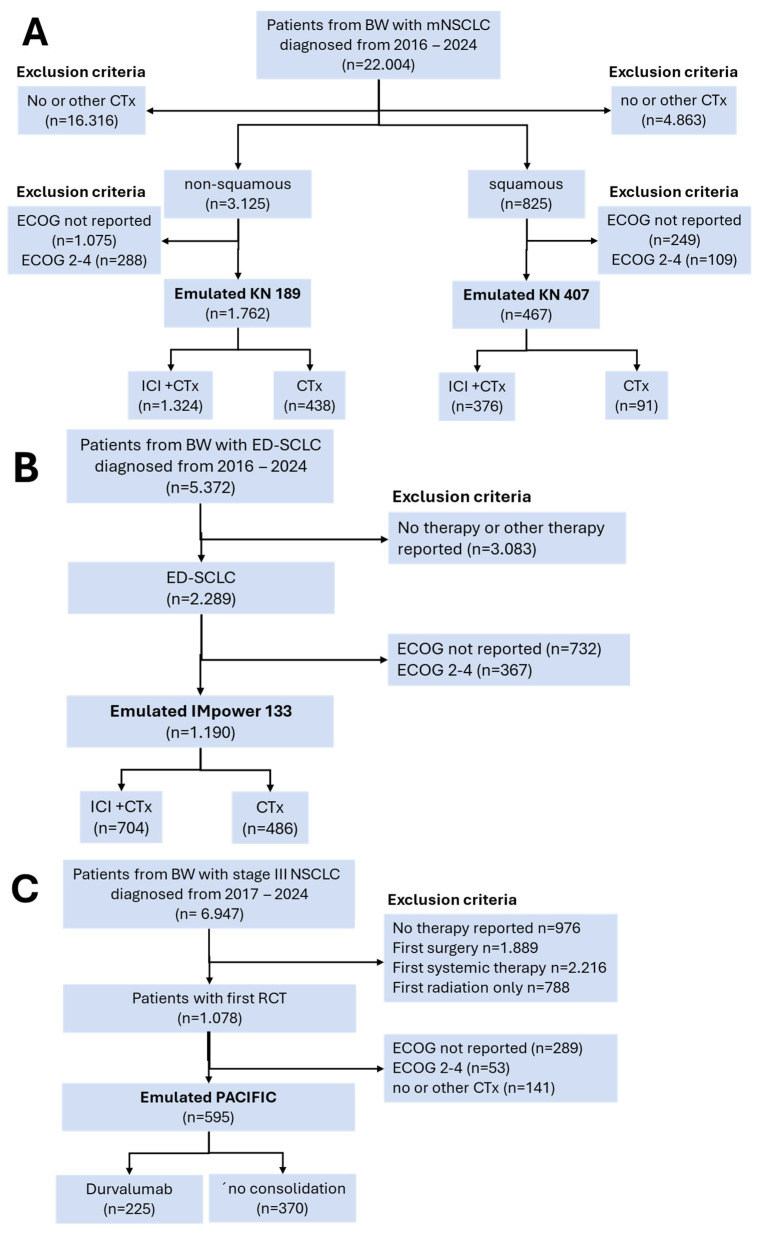
CONSORT diagram of patient selection. CTx refers to platinum-based chemotherapy; CITx indicates chemo-immunotherapy, defined as CTx plus pembrolizumab for mNSCLC or CTx plus atezolizumab for ED-SCLC. (**A**) eKN189 and eKN407; (**B**) eIMP133; and (**C**) ePACIFIC.

Details of the patient selection and cohort definitions are illustrated in Figure 1.

### 2.4. Time-Zero and Endpoints

Time-zero was defined as the initiation date of systemic therapy for eKN189, eKN407 and eIMP133, and as the end date of radiotherapy for ePACIFIC.

The primary endpoint was the overall survival (OS), defined as the time from time-zero to death from any cause, ascertained through registry follow-up. The secondary endpoints included the objective response rate (ORR), derived from the registry tumor status field documented in routine clinical update reports. The tumor response was categorized as: (i) complete remission, (ii) partial remission, (iii) stable disease, or (iv) progression. The ORR was defined as the proportion of patients achieving complete or partial remission. Because a registry-based response assessment does not fully correspond to a RECIST-defined radiologic response evaluation, the ORR analyses should be interpreted as a pragmatic real-world approximation.

### 2.5. Emulation of Randomization Using Propensity Score Weighting

Because the treatment assignment in registry data is non-randomized, we emulated a random treatment allocation using propensity-score-based methods. For each emulation, a propensity score was estimated representing the probability of receiving the experimental versus the control strategy based on baseline covariates measured prior to time-zero. The covariates were selected based on clinical relevance, prognostic importance, and availability in the registry, and included the age, sex, ECOG performance status, disease stage, and chemotherapy (CTx) backbone where applicable.

Inverse probability of the treatment weighting (IPTW) was applied to estimate the average treatment effects in a weighted pseudo-population with balanced baseline covariates, consistent with established recommendations for causal inference in observational studies [25,26]. The covariate balance before and after weighting was assessed using the standardized mean differences (SMDs), with values < 0.1 considered indicative of an acceptable balance [25]. Graphical balance diagnostics using Love plots confirmed substantial reduction of baseline imbalances after weighting across all emulations (Supplementary Figure S1a–d).

The weighted analyses were interpreted as intention-to-treat-like estimates under the assumptions of conditional exchangeability, positivity, and correct model specification [24]. As with all observational analyses, residual confounding due to unmeasured variables cannot be excluded.

### 2.6. Missing Data

Missingness was assessed descriptively for key baseline variables. Patients with a missing ECOG status were excluded because ECOG 0–1 formed part of the eligibility definition. PD-L1 data were incomplete, and used only in exploratory subgroup analyses where available. Response analyses were limited to patients with available follow-up documentation. No multiple imputation was performed, as missingness primarily affected trial-specific variables not required for cohort construction or primary survival analyses.

### 2.7. Sample Size Considerations

No formal a priori power calculations were performed due to the retrospective, registry-based design. The sample size was determined by the number of patients meeting trial eligibility criteria in the registry.

For most emulations, the cohort sizes were comparable to or exceeded the original RCTs, while in some cases, a treatment arm included fewer patients. Analyses were still conducted, acknowledging that smaller samples may reduce precision of the effect estimates.

### 2.8. Statistical Analysis

The baseline characteristics were summarized descriptively in the unweighted population and compared between the treatment groups using Fisher’s exact test or the chi-square test, as appropriate.

The OS was estimated using weighted Kaplan–Meier methods, and compared using a weighted log-rank test. The treatment effects on the overall survival were estimated using weighted Cox proportional hazard models, yielding hazard ratios (HRs) and 95% CIs. Subgroup analyses were conducted within weighted populations using stratified Cox models and should be interpreted as exploratory and hypothesis-generating only.

The median follow-up time was estimated using the reverse Kaplan–Meier method. All statistical analyses were conducted in R version 4.1.1 (https://www.r-project.org/). Propensity score estimation and weighting were conducted using established R packages for causal inference (cobalt, mice, WeightIt, survey), and survival analyses were performed using the survival (version 3.8-6) and survminer (version 0.5-2) packages.

### 2.9. Ethical Considerations

In accordance with the Baden-Württemberg State Cancer Registry Act, physicians and dentists are required to inform patients about the notification of their cancer diagnosis to the registry. Patients have the right to object in writing to the further processing of their identifiable data by the trust center, the clinical state registry, or the epidemiological cancer registry. All data used in this study were provided in pseudonymized form by the Clinical Cancer Registry of Baden-Württemberg. No identifiable patient data were available to the researchers, and no additional data sources were used. All analyses were conducted using aggregated and de-identified data. Consequently, ethical review and approval were not required. The study complies with the principles outlined in the Declaration of Helsinki.

## 3. Results

### 3.1. Cohort Derivation and Baseline Features

A total of 4015 patients from the BWCR met the eligibility criteria for four emulated cohorts (eKN189: 1762, eKN407: 467, eIMP133: 1190 and ePACIFIC: 595). The patient flow is illustrated in Figure 1. The most frequent reasons for exclusion were an ECOG > 1 or missing, and initiation of therapies not defined in the original RCT protocols.

The baseline demographic and clinical characteristics are summarized in Table 1. The median age ranged from 65 to 66 years, comparable to the respective RCT populations. The proportion of male patients was slightly lower than in the trials. Across all cohorts, approximately 60% of the patients had an ECOG performance status of 1.

### 3.2. Emulated KEYNOTE-189: Pembrolizumab Plus Chemotherapy in Non-Squamous mNSCLC

The eKN189 cohort included 1762 patients, of whom 1324 received pembrolizumab plus CTx and 438 CTx alone. The median age was 65 years, 57% were male and the histology was adenocarcinoma (≈95%). In the unadjusted cohort, imbalances were observed in the chemotherapy backbone and metastatic stage (Table 1).

After inverse probability of the treatment weighting, the baseline characteristics were well balanced (all SMD < 0.1). In the weighted pseudo-population, the age, sex, ECOG performance status, platinum backbone, and prevalence of brain and liver metastases were comparable across treatment groups, supporting the validity of subsequent comparative analyses.

#### 3.2.1. eKN189: Tumor Response

The tumor response was evaluated in 1345 patients with ≥3 months of follow-up. The ORR was higher with pembrolizumab plus chemotherapy than with chemotherapy alone (41.3% vs. 26.6%; *p* < 0.001). Complete responses occurred in 2.3% vs. 2.2%, and partial responses in 38.9% vs. 25.0%, respectively. The median time to response was ~3 months in both groups. Progression without a prior response was more frequent with chemotherapy alone (60.3% vs. 45.6%).

#### 3.2.2. eKN189: Overall Survival

**Figure 2:**
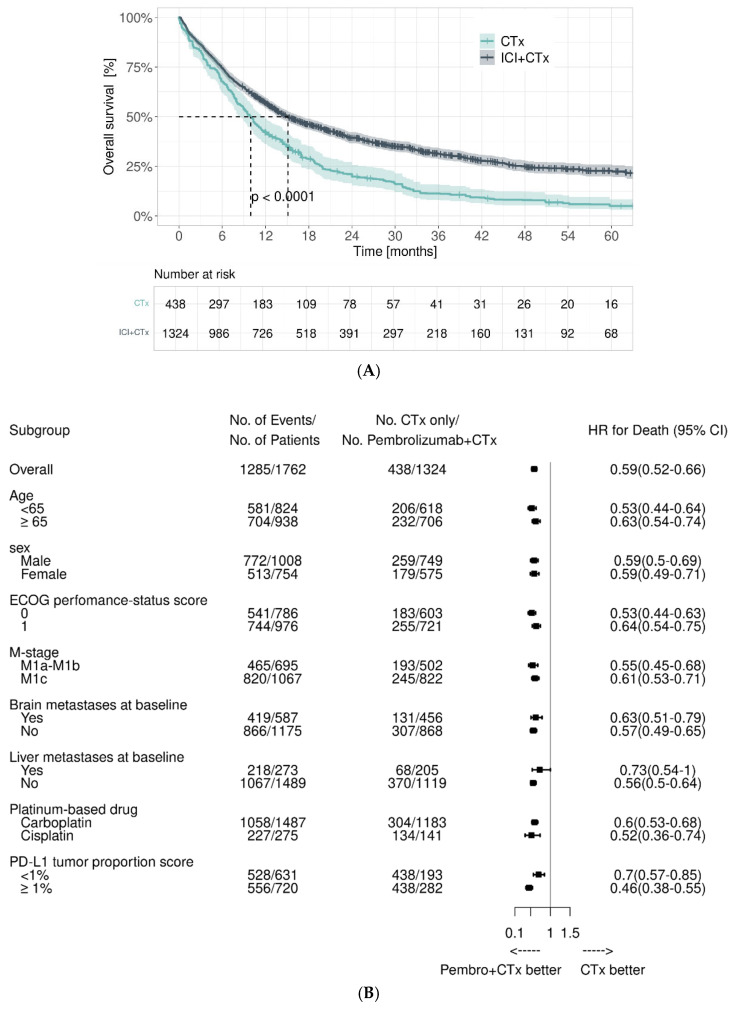
(**A**) Propensity-score-weighted Kaplan–Meier curves for OS comparing pembrolizumab plus chemotherapy (CITx) versus chemotherapy alone (CTx) in the eKN189 cohort. (**B**) Subgroup analysis of OS using weighted Cox proportional hazard models. HRs and 95% CIs are shown for selected key subgroups. HRs < 1 favor pembrolizumab plus chemotherapy.

The median follow-up was 42.6 months. In the propensity-score-weighted population, treatment with pembrolizumab in combination with pemetrexed and platinum chemotherapy was associated with a significantly longer median OS compared with CTx alone: 15.1 vs. 9.95 months (*p* < 0.0001, weighted log-rank test). The twelve-month OS rate was 57% for ICI + CTx compared with 42% for CTx. At five years, the OS rate was 22.7% (95% CI, 19.9–25.8%) in the CITx group versus 4.7% (95% CI, 2.8–7.9%) in the CTx group. The weighted Kaplan–Meier curves indicated an early and sustained survival benefit for the ICI + CTx group (Figure 2A).

The weighted Cox models confirmed OS benefits across the subgroups, including the age (<65 vs. ≥65), sex, ECOG (0 vs. 1), and metastatic burden (overall HR 0.59, 95% CI 0.52–0.62) groups (Figure 2B). By the PD-L1 expression, the HR was 0.7 (95% CI, 0.57–0.85) for TPS < 1% and 0.46 (95% CI, 0.38–0.74) for ≥1%. A relative 8% difference in the HRs was observed for cisplatin versus carboplatin (HR, 0.52 vs. 0.60), suggesting a potential advantage for cisplatin-based regimens.

#### 3.2.3. Comparison with KEYNOTE-189

The adjusted eKN189 cohort was broadly comparable to the KEYNOTE-189, with a median age of 65 years. The sex distribution was more balanced in the real-world cohort, whereas brain metastases were more common than in the trial (33% vs. 17%). Liver metastases were similar, while cisplatin use was lower in the real-world cohort (10.6%) compared with KEYNOTE-189 (28%).

The OS outcomes were directionally consistent with the RCT. A survival plateau in the ICI + CTx group mirrored the trial pattern. The HRs overall and across key subgroups aligned with the RCT and fell within the corresponding confidence intervals. The median OS for the combination was slightly lower in the real-world cohort; the 5-year OS was comparable (22% vs. 19.4%) and within the 95% CI of the trial. The larger real-world sample yielded narrower confidence intervals, enhancing precision of the subgroup estimates.

### 3.3. Emulated KEYNOTE-407: Pembrolizumab Plus Chemotherapy in Squamous mNSCLC

The eKN407 cohort included 467 patients: 376 received pembrolizumab plus chemotherapy and 91 received chemotherapy alone (Table 2). The patients were slightly older than in the RCT (median, 68 vs. 65 years), with more females (26.8% vs. 16.6%) and a higher prevalence of brain metastases (14.8% vs. 7.7%). Nab-paclitaxel was used more frequently than in the trial (60.0% vs. 39.9%). After inverse probability weighting, the baseline characteristics were well balanced (all SMD < 0.1).

#### 3.3.1. eKN407: Tumor Response

Clinical update reports were available for 340 patients. The ORR was numerically higher with pembrolizumab plus chemotherapy than chemotherapy alone (44.4% vs. 35.4%, *p* = 0.086). Complete responses occurred in 3.3% vs. 3.1% and partial responses in 45.8% vs. 32.3%, respectively. The median time to response was approximately 3 months in both groups. Progression without a documented response was more frequent with CTx alone (58.5% vs. 45.8%).

#### 3.3.2. eKN407: Overall Survival

**Figure 3:**
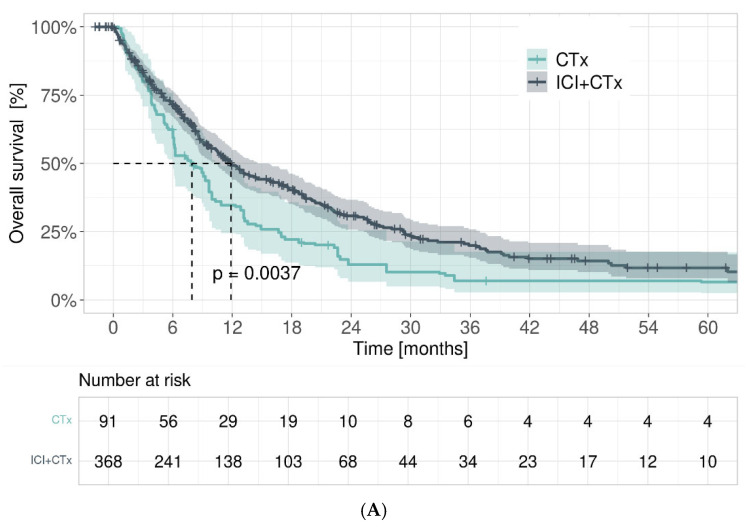

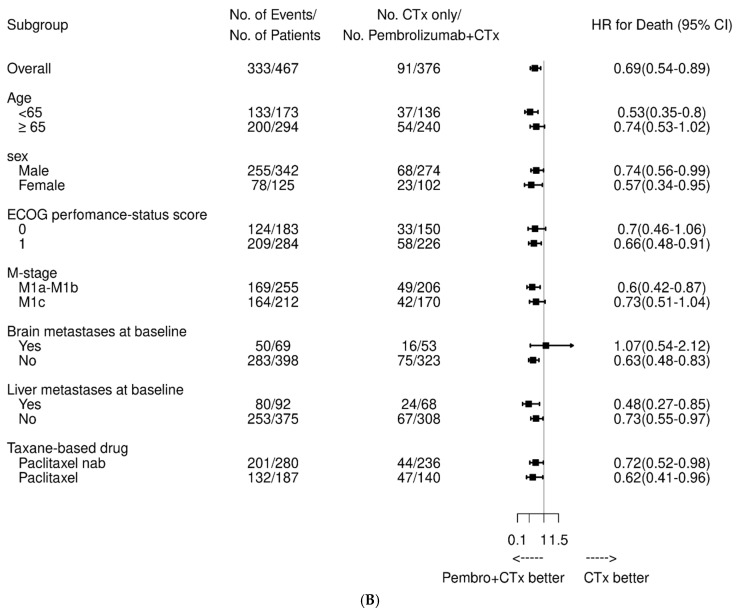
(**A**) Propensity score-weighted Kaplan–Meier curves for OS comparing pembrolizumab plus chemotherapy (CITx) versus chemotherapy alone (CTx) in the eKN407 cohort. (**B**) Subgroup analysis of OS using weighted Cox proportional hazard models. HRs and 95% CIs are shown for selected key subgroups. HRs < 1 favor pembrolizumab plus chemotherapy.

The median follow-up was 35.6 months. Pembrolizumab with chemotherapy significantly reduced the risk of death compared to chemotherapy alone (HR, 0.69; 95% CI, 0.54–0.89; Figure 3).

The mOS was 11.9 months (95% CI, 10.4–15.7) with CITx vs. 7.9 months (95% CI, 6.0–10.9) with CTx (Figure 3A). The five-year OS was 11.8% (95% CI, 7.8–17.7%) versus 6.6% (95% CI, 2.4–17.5%), with a visible survival plateau in the CITx group.

Across all predefined subgroups, the combination therapy showed a survival advantage, though in some subgroups, the statistical significance was not reached, likely reflecting the smaller sample size, particularly in the chemotherapy-only cohort (Figure 3B).

### 3.4. Emulated IMpower-133: Atezolizumab Plus Chemotherapy in ES-SCLC

The eIMP133 cohort included 1.190 patients, more than twice the size of each arm of the original RCT (486 CITx; 704 CTx). The median age and sex distribution were similar among groups. Some baseline differences were observed for the ECOG performance status, with more ECOG 0 patients in the CITx group, and for the disease burden, including brain and liver metastases (Table 3).

After propensity score weighting, the baseline characteristics were well balanced, with all the standardized mean differences being below the accepted thresholds. Key variables—including the age, sex, ECOG status, metastatic stage, and brain and liver metastases—showed minimal residual imbalances, indicating good comparability between treatment groups.

#### 3.4.1. eIMP133: Tumor Response

Among the 947 patients with available clinical update reports, the ORR was significantly higher with atezolizumab plus chemotherapy than with chemotherapy alone (51.7% vs. 43.6%; *p* = 0.05).

In the CITx group, 2% achieved a complete and 49.7% achieved a partial response, compared with 1.3% and 42.3% in the CTx group. The median time to response was roughly 3 months in both groups. Progression without a documented response was more frequent in the CTx group (45.7% vs. 43.4%).

#### 3.4.2. eIMP133: Overall Survival

**Figure 4:**
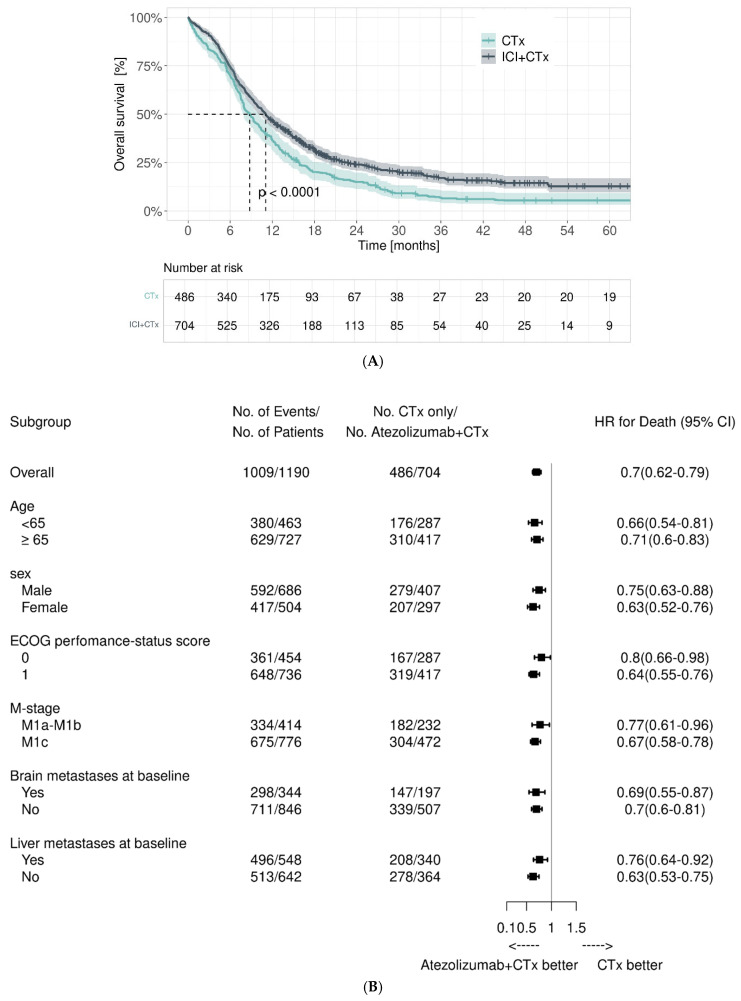
(**A**) Propensity-score-weighted Kaplan–Meier curves for OS comparing atezolizumab plus chemotherapy (CITx) versus chemotherapy alone (CTx) in the eIMP133 cohort. (**B**) Subgroup analysis of OS overall survival using weighted Cox proportional hazards models. HRs and 95% CIs are shown for selected key subgroups. HRs < 1 favor pembrolizumab plus chemotherapy.

In the eIMP133 cohort, the median follow-up was 34.6 months. The addition of atezolizumab to platinum-etoposide CTx was associated with an improved OS (HR, 0.7; 95% CI, 0.62–0.79; Figure 4). The median OS was 11.1 months (95% CI, 10.0–12.1) with CITx versus 8.8 months (95% CI, 7.9–10.1) with CTx alone (Figure 4A). The five-year OS was 12.8% (95% CI, 9.6–17.0%) versus 5.3% (95% CI, 3.1–9.0%), respectively. The CITx survival curve showed a plateau, suggesting a durable benefit in a subset of patients.

The survival benefit from atezolizumab-based therapy was observed across all predefined subgroups (Figure 4B), including patients with brain metastasis (HR, 0.69; 95% CI, 0.55–0.87) and those <65 years (HR, 0.66; 95% CI, 0.54–0.81), with similar effects in older patients (HR, 0.71; 95% CI, 0.6–0.83). The benefit was more pronounced in patients with M1c disease (HR, 0.67; 95% CI, 0.58–0.78) than in those with M1a/M1b stages (HR, 0.77; 95% CI, 0.61–0.96).

#### 3.4.3. Comparison with the IMpower133 Trial

Baseline characteristics of the eIMP133 cohort were generally comparable to those of the IMpower133 trial. The median age was slightly higher in the real-world cohort (66 vs. 64 years), and brain metastases were more common (30% vs. 8.5%).

The treatment effects across the subgroups were directionally consistent with the trial. However, some subgroups that were not statistically significant in the RCT—particularly patients < 65 years and those with brain or liver metastases—showed significant survival benefits in the real-world analysis, likely reflecting the larger sample size.

### 3.5. Emulated PACIFIC: Durvalumab Consolidation in Unresectable Stage III NSCLC

The ePACIFIC cohort comprised 595 patients with unresectable stage III NSCLC treated with definitive radiochemotherapy between 2017 and 2024; 225 received durvalumab consolidation and 370 did not (Table 4). The median age was 66 years, 70% were male, and 48.3% had squamous histology. The median radiation dose was 63 Gy (range, 24–72 Gy) in the durvalumab group and 50 Gy (range, 12–77 Gy) in the no-consolidation group. Concurrent chemotherapy was carboplatin-based in 33.4% and cisplatin-based in 66.6%. After PSW, the baseline characteristics were well balanced (SMD < 0.1).

#### 3.5.1. ePACIFIC: Tumor Response

Follow-up response assessments were available for 472 patients. The response rate was significantly higher with durvalumab consolidation compared with no consolidation (56.7% vs. 49.7%; *p* = 0.004). In the durvalumab group, 13.9% patients achieved a complete response and 42.8% a partial response, whereas in the non-durvalumab group, 13.7% patients had a complete response and 42.8% a partial response.

Progression without a prior documented response occurred in 39.4% of patients in the non-durvalumab group versus 25.6% in the durvalumab group. The median time to progression was 8.1 months with durvalumab compared with 5.9 months without. Among the responders, the median duration of response was 9.4 months with durvalumab versus 5.8 months without consolidation therapy.

#### 3.5.2. ePACIFIC: Overall Survival

**Figure 5:**
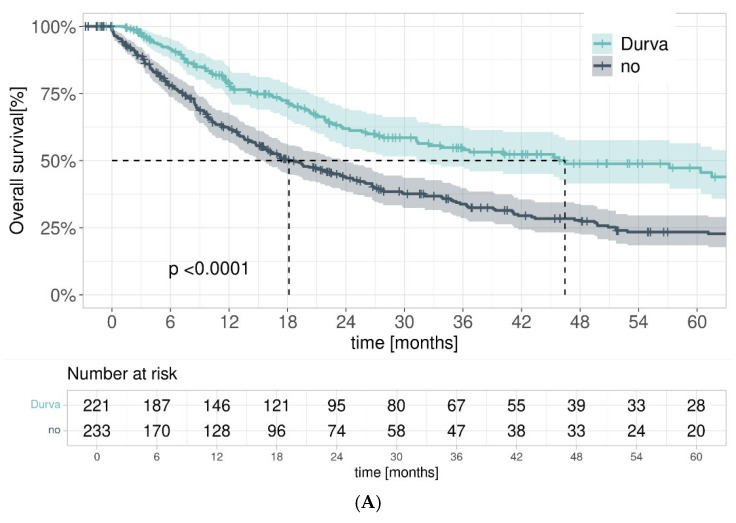

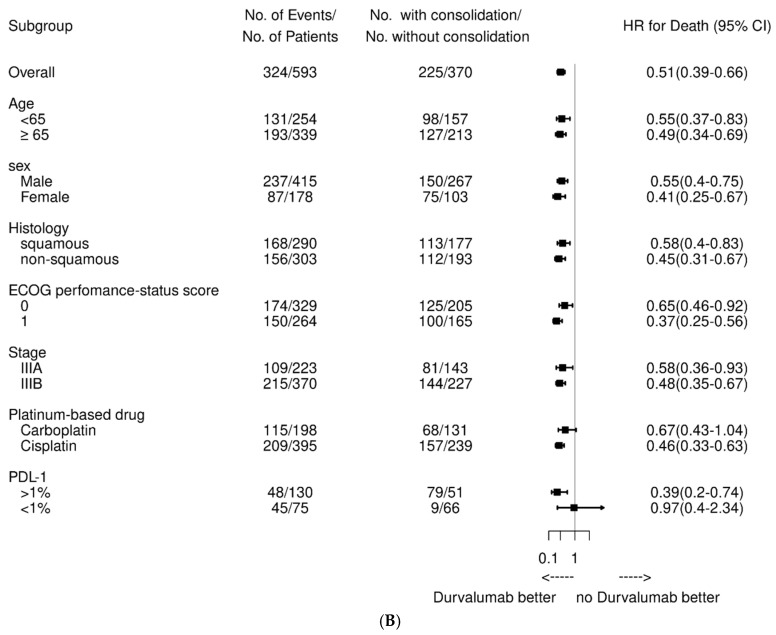
(**A**) Propensity-score-weighted Kaplan–Meier curves for OS comparing durvalumab consolidation versus no consolidation in the ePACIFIC cohort. (**B**) Subgroup analysis of OS using weighted Cox proportional hazard models. HRs and 95% CIs are shown for selected key subgroups. HRs < 1 favor pembrolizumab plus chemotherapy.

The median follow-up was 37.7 months. The median OS was 46.4 months (95% CI, 31.4–65.1) with durvalumab consolidation versus 18.1 months (95% CI, 15.6–23.8) without (Figure 5A). The five-year OS was 47.3% (95% CI, 39.5–56.5%) versus 23.4% (95% CI, 18.5–29.6%). Durvalumab was associated with a significant reduction in the risk of death (HR, 0.51; 95% CI, 0.39–0.66; Figure 5B).

A survival benefit was observed across most predefined subgroups, except for patients with PD-L1 < 1%, where the case numbers were very small. The benefit was similar in younger and older patients (≥65 years: HR, 0.49; 95% CI, 0.34–0.69). The outcomes also differed by chemotherapy backbone, with a stronger effect after cisplatin-based chemoradiotherapy (HR, 0.46; 95% CI, 0.40–0.63) than after carboplatin-based treatment (HR, 0.67; 95% CI, 0.43–1.04).

#### 3.5.3. Comparison with the PACIFIC Trial

The baseline characteristics were broadly comparable to the PACIFIC population, although stage IIIB disease was more common in our cohort (63.2% vs. 44.5%). In contrast to the PACIFIC trial, we observed a similar survival benefit in both younger and older patients, whereas the trial did not show a clear advantage in older patients. Similar to the trial, no clear survival advantage was observed after carboplatin-based chemoradiotherapy.

## 4. Discussion

In this population-based registry study applying a targeted trial emulation framework with PSW, we found that the survival and response benefits of CPI-based therapies reported in the landmark KEYNOTE-189, KEYNOTE-407, IMpower133, and PACIFIC trials were largely reproducible in routine clinical practice. To our knowledge, this is the first large-scale study to emulate four pivotal lung cancer RCTs within a single population-based registry using a unified methodological framework.

In non-squamous mNSCLC (eKN189), the 5-year OS with pembrolizumab plus chemotherapy reached 22.7%, closely matching the trial [5]; in squamous mNSCLC (eKN407), it was 11.8%, consistent with the original study [8]. ES-SCLC patients treated with atezolizumab plus chemotherapy achieved a 5-year OS of 12.8%, aligning with IMpower133 [11], and durvalumab consolidation in unresectable stage III NSCLC yielded 47.3%, consistent with PACIFIC [14]. Across all cohorts, the OS curves in CPI-containing arms demonstrated characteristic long-term survival plateaus, reflecting a durable benefit in a subset of patients, as observed in the original randomized trials. Notably, long-term survivors in our registry did not appear to differ substantially from the overall trial-like populations with regard to their age, ECOG performance status, or metastatic burden, although dedicated analyses were beyond the scope of the present study.

The objective response rates were similarly consistent with the trial results: in eKN189, the ORR was 41.6% vs. 29.6% for chemotherapy alone; in eKN407, it was 44.8% vs. 31.5%; in eIMP133, it was 51.7% vs. 43.1%; and in ePACIFIC, it was 56.6% vs. 47.1% (CPI vs. control). These effects remained directionally consistent across clinically relevant subgroups, including older patients, those with brain metastases, and patients receiving differing chemotherapy backbones. The subgroup analyses should be interpreted as exploratory and hypothesis-generating only.

The target trial emulation framework enabled explicit alignment of the eligibility criteria, treatment strategy, time-zero, follow-up, and endpoint definitions with those of the original RCTs. Together with propensity score weighting, this balanced the measured baseline covariates among the treatment groups. This design addresses common sources of bias in observational oncology research, including an immortal time bias and confounding by indication, thereby improving the comparability between register-based estimates and randomized evidence [21,22,23]. However, causal interpretation remains conditional on assumptions of exchangeability, positivity, and correct model specification, and residual confounding from unmeasured variables cannot be excluded.

The baseline characteristics in our real-world cohorts were broadly comparable to those of the trial populations, including the ECOG performance status, median age, and sex distribution. Nonetheless, some clinically relevant differences were observed, such as a higher prevalence of brain metastases or more frequent use of cisplatin in certain chemotherapy backbones. Importantly, despite these differences, the survival and response benefits of CPI-based therapies remained consistent with the findings from the corresponding RCTs, suggesting that CPI efficacy is transferable to routine-care populations selected according to trial-like criteria. At the same time, because our emulations were intentionally restricted to ECOG 0–1 patients, these findings should not be interpreted as representative of the full real-world population.

Differences in the chemotherapy backbones were notable. In eKN189, an 8% relative difference in the hazard ratios between the cisplatin and carboplatin subgroups (HR, 0.52 vs. 0.6) suggested a potential survival advantage with cisplatin. The observed trend mirrors findings from KEYNOTE-189. Similarly, in ePACIFIC, patients receiving cisplatin concurrent with radiotherapy followed by durvalumab exhibited a pronounced OS benefit (HR, 0.46), whereas those treated with carboplatin showed no significant benefit (HR, 0.67; 95% CI, 0.46–1.04). These observations likely reflect both regimen effects and residual treatment-selection factors and should therefore be interpreted cautiously.

Previous real-world studies have generally confirmed the long-term benefits of combining CPIs with chemotherapy, particularly in squamous and non-squamous mNSCLC populations [19,20]. More recently, the CORRELATE study [28] further demonstrated improved outcomes with first-line immunotherapy-based approaches in metastatic NSCLC under real-world conditions. However, most of these analyses describe real-world treatment patterns, frequently combining different immunotherapy regimens (e.g., pembrolizumab monotherapy, pembrolizumab plus chemotherapy, or nivolumab/ipilimumab combinations) without isolating a trial-like cohort [18,29]. Consequently, direct comparisons with RCT outcomes remain limited.

To our knowledge, only a few studies have attempted to emulate RCT outcomes using real-world registry-based data. For example, KEYNOTE-189 [30] and PACIFIC [31] have been explored in this context. The analysis of KEYNOTE-189 [30] provided a preliminary signal in a larger population, but was not confined to the trial’s strict inclusion criteria and did not fully replicate the exact trial setting. The PACIFIC [31] analysis included an extremely small cohort (83 patients), which strongly limits interpretability.

In contrast, our study emulated four landmark lung cancer trials within a single population-based registry using a consistent methodological framework and large cohorts, offering key advantages: a large, population-based sample (n = 4015), a long follow-up (median of >35 months), and a robust subgroup evaluation, enabling a more systematic assessment of the reproducibility across multiple disease settings. Recent methodological work further supports the validity and scalability of target trial emulation approaches in oncology and registry science [22,23,24,25,26,27].

Several limitations should be acknowledged. First, although PSW improved the balance among the treatment groups, residual confounding from unmeasured variables cannot be excluded. Important trial-specific variables—such as adverse events, detailed comorbidities, and smoking status—were not systematically captured. Second, the PD-L1 status was available, but incomplete, limiting interpretability of biomarker-stratified analyses. Third, the tumor response assessment in the registry does not fully correspond to RECIST-defined ORRs and relies on clinical documentation rather than a standardized radiologic evaluation. Finally, variation in the systemic therapy regimens—including the dosing, duration, and supportive care—across centers may have influenced the outcomes and limited the strict protocol-level comparability.

## 5. Conclusions

Despite important limitations inherent to observational registry data, the large population-based cohort, long follow-up, and explicit trial emulation framework support the finding that the benefits of checkpoint-inhibitor-based therapies observed in pivotal randomized trials can be closely approximated in trial-eligible routine-care populations. To our knowledge, this study represents the first large-scale application of a unified target trial emulation approach across multiple landmark lung cancer RCTs, demonstrating the feasibility of systematically reproducing trial-like analyses using high-quality registry data. These findings support the transportability of pivotal trial results to comparable real-world patient populations and highlight the growing value of population-based cancer registries as complementary infrastructures for external validation, comparative effectiveness research, and evidence generation in thoracic oncology.

## Figures and Tables

**Table 1:** Baseline characteristics of patients in the emulated KN-189 cohort before and after propensity score weighting. Values are presented as n (%) or median (range). Standardized mean differences (SMDs) are shown to quantify the covariate balance between treatment groups before and after propensity score weighting; SMD values < 0.1 indicate an adequate balance. Adjusted values represent weighted estimates and may therefore be non-integer.

	Unadjusted Emulated KN189-Cohort			Adjusted Emulated KN189-Cohort		
	CTx	CTx + ICI	SMD	CTx	CTx + ICI	SMD
Characteristic						
n	438 (24.9)	1324 (75.1)		438 (24.9)	1324 (75.1)	
Age-median (range)	65.0 [31.0, 84.0]	65.0 [29.0, 90.0]	0.062	65.0 [31.0, 84.0]	65.0 [29.0, 90.0]	0.001
Sex			0.052			
m	259 (59.1)	749 (56.6)		247.8 (56.6)	749 (56.6)	<0.001
w	179 (40.9)	575 (43.4)		190.2 (43.4)	575 (43.4)	
ECOG			0.076			0.001
0	183 (41.8)	603 (45.5)		199.3 (45.5)	603 (45.5)	
1	255 (58.2)	721 (54.5)		238.7 (54.5)	721 (54.5)	
metastatic stage			0.150			0.013
M1a	82 (20.2)	250 (20.0)		81.2 (19.5)	250 (20.0)	
M1b	97 (24.0)	227 (18.1)		75.1 (18.0)	227 (18.1)	
M1c	226 (55.8)	775 (61.9)		260.2 (62.5)	775 (61.9)	
metastasis location						
BRA	131 (29.9)	456 (34.4)	0.097	129.2 (29.5)	456 (34.4)	0.1
HEP	68 (15.5)	205 (15.5)	0.001	65.8 (15.0)	205 (15.5)	0.013
Platinum CTx type....			0.509			0.002
Carboplatin	304 (69.4)	1183 (89.4)		391.1 (89.3)	1183 (89.4)	
Cisplatin	134 (30.6)	141 (10.6)		46.9 (10.7)	141 (10.6)	

**Table 2:** Baseline characteristics of patients in the emulated KN-407 cohort before and after propensity score weighting. Values are presented as n (%) or median (range). Standardized mean differences (SMDs) are shown to quantify the covariate balance between treatment groups before and after propensity score weighting; SMD values < 0.1 indicate an adequate balance. Adjusted values represent weighted estimates and may therefore be non-integer.

	Unadjusted Emulated KN407-Cohort			Adjusted Emulated KN407-Cohort		
	CTx	CTx + ICI	SMD	CTx	CTx + ICI	SMD
Characteristic						
n (%)	91 (19.5)	376 (80.5)		91. (19.5)	376.0 (80.5)	
Age-median (range)	66 [27, 87]	68 [42, 92]	0.078	66 [27, 87]	68 [42, 92]	0.007
Sex			0.042			0.008
m	68 (74.7)	274 (72.9)		66.6 (73.2)	274.0 (72.9)	
w	23 (25.3)	102 (27.1)		24.4 (26.8)	102.0 (27.1)	
ECOG			0.075			0.001
0	33 (36.3)	150 (39.9)		36.3 (39.9)	150.0 (39.9)	
1	58 (63.7)	226 (60.1)		54.7 (60.1)	226.0 (60.1)	
metastatic stage			0.057			0.043
M1a	25 (29.4)	97 (26.9)		23.9 (28.0)	97.0 (26.9)	
M1b	23 (27.1)	103 (28.6)		22.8 (26.7)	103.0 (28.6)	
M1c	37 (43.5)	160 (44.4)		38.6 (45.3)	160.0 (44.4)	
metastasis location						
BRA	16 (17.6)	53 (14.1)	0.096	13.0 (14.3)	53.0 (14.1)	0.002
HEP	24 (26.4)	68 (18.1)	0.200	16.3 (17.9)	68.0 (18.1)	0.006
Taxane			0.293			0.004
paclitaxel	47 (51.6)	140 (37.2)		34.0 (37.3)	140.0 (37.2)	
paclitaxel nab	44 (48.4)	236 (62.8)		57.0 (62.7)	236.0 (62.8)	

**Table 3:** Baseline characteristics of patients in the emulated Impower133 cohort before and after propensity score weighting. Values are presented as n (%) or median (range). Standardized mean differences (SMDs) are shown to quantify the covariate balance between treatment groups before and after propensity score weighting; SMD values < 0.1 indicate an adequate balance. Adjusted values represent weighted estimates and may therefore be non-integer.

	Unadjusted Emulated Impower133-Cohort			Adjusted Emulated Impower 133-Cohort		
	CTx	CTx + ICI	SMD	CTx	CTx + ICI	SMD
Characteristic						
n	486 (40.8)	704 (59.2)		486 (40.8)	704 (59.2)	
Age-median (range)	65.0 [31.0, 84.0]	65.0 [29.0, 90.0]	0.094	66.0 [39.0, 87.0]	66.0 [32.0, 87.0]	0.002
Sex			0.008			0.001
m	279 (57.4)	407 (57.8)		197.8 (40.7)	287 (40.8)	
w	207 (42.6)	297 (42.2)		288.2 (59.3)	417 (59.2)	
ECOG			0.133			0.001
0	167 (34.4)	287 (40.8)		197.8 (40.7)	287 (40.8)	
1	319 (65.6)	417 (59.2)		288.2 (59.3)	417 (59.2)	
metastatic stage			0.114			0.026
M1a	59 (13.1)	66 (9.9)		42.0 (9.3)	66 (9.9)	
M1b	114 (25.2)	159 (23.8)		111.6 (24.7)	159 (23.8)	
M1c	279 (61.7)	443 (66.3)		298.2 (66.0)	443 (66.3)	
metastasis location						
BRA	147 (30.2)	197 (28.0)	0.050	155.3 (32.0)	197 (28.0)	0.087
HEP	208 (42.8)	340 (48.3)	0.111	211.8 (43.6)	340 (48.3)	0.095

**Table 4:** Baseline characteristics of patients in the emulated PACIFIC cohort before and after propensity score weighting. Values are presented as n (%) or median (range). Standardized mean differences (SMDs) are shown to quantify the covariate balance between treatment groups before and after propensity score weighting; SMD values < 0.1 indicate an adequate balance. Adjusted values represent weighted estimates and may therefore be non-integer.

	Unadjusted Emulated PACIFIC-Cohort			Adjusted Emulated PACIFIC-Cohort		
	Durvalumab	No Durvalumab	SMD	Durvalumab	No Durvalumab	SMD
Characteristic						
n (%)	225 (37.8)	370 (62.2)		225.0	239.0	
Age-median (range)	65.0 [44.0, 84.0]	66.5 [39.0, 93.0]	0.008	65.0 [44.0, 84.0]	66.0 [39.0, 93.0]	0.006
Sex			0.119			0.058
m	150 (66.7)	267 (72.2)		150.0 (66.7)	165.8 (69.4)	
w	75 (33.3)	103 (27.8)		75.0 (33.3)	73.2 (30.6)	
ECOG			0.003			0.002
0	125 (55.6)	205 (55.4)		125.0 (55.6)	133.1 (55.7)	
1	100 (44.4)	165 (44.6)		100.0 (44.4)	105.9 (44.3)	
stage			0.055			0.032
IIIa	224 (37.6)	81 (36.0)		81.0 (36.0)	89.8 (37.6)	
IIIb	371 (62.4)	144 (64.0)		144.0 (64.0)	149.2 (62.4)	
radiation dose-median	63	60	0.26	63.0	62.0	0.1
platin			0.11			0.029
carboplatin	68 (30.2)	131 (35.4)		68.0 (30.2)	79.5 (33.2)	
cisplatin	157 (69.8)	239 (64.6)		157.0 (69.8)	159.6 (66.8)	

## Data Availability

The data that support the findings of this study are available from the corresponding author upon request. The data are not publicly available due to privacy or ethical restrictions.

## Supplementary Materials

The following supporting information can be downloaded at: https://www.mdpi.com/article/10.3390/cancers18111754/s1, Table S1: Target trial emulation framework and alignment of pivotal randomized controlled trials with registry-based emulations; Figure S1a: Covariate balance in the eKN189 cohort; Figure S1b: Covariate balance in the eKN407 cohort; Figure S1c: Covariate balance in the eIMP133 cohort; Figure S1d: Covariate balance in the ePACIFIC cohort.

## Abbreviations

The following abbreviations are used in this manuscript:

BW	Federal State of Baden-Württemberg, Germany
BWCR	Cancer Registry of Baden-Württemberg
CI	Confidence interval
CTx	Chemotherapy
CITx	Chemoimmunotherapy
CPI	Checkpoint inhibitor
eIMP133	Emulated IMpower133
eKN189	Emulated KEYNOTE-189
eKN407	Emulated KEYNOTE-407
ePACIFIC	Emulated PACIFIC
HR	Hazard ratio
ICI	Immune checkpoint inhibitor
mNSCLC	Metastatic non-small cell lung cancer
mOS	Median overall survival
NSCLC	Non-small cell lung cancer
ORR	Overall response rate
OS	Overall survival
Pembro + CTx	Pembrolizumab plus chemotherapy
PSW	Propensity score weighting
RCT	Randomized controlled trial
RWD	Real-world data
SMD	Standardized mean difference
