# Supplementary material for: Real-World Emulation of Landmark Lung Cancer Trials: A Registry-Based Reconstruction of KN189, KN407, IMpower133, and PACIFIC
Source: Cancers (Basel). 2026 May 27;18(11):1754. doi: 10.3390/cancers18111754 (PMC13255646; doi:10.3390/cancers18111754)
Supplement: Supplementary file 1 [file cancers-18-01754-s001.zip › cancers-4313362-supplementary.pdf]

SUPPLEMENTARY MATERIAL

This Supplemental Material provides additional analyses that support the findings of our main manuscript.

Table S1 summarizes the target trial emulation framework applied to replicate four pivotal randomized controlled trials (KEYNOTE-189, KEYNOTE-407, IMpower133, and PACIFIC) using population-based cancer registry data. For each study, eligibility criteria, treatment strategies, time-zero definitions, endpoints, and key protocol deviations were aligned as closely as possible between the original randomized trials and their corresponding emulated cohorts (eKN189, eKN407, eIMP133, and ePACIFIC). Registry-derived approximations were used where direct clinical trial variables (e.g., RECIST-based assessments or PD-L1 status completeness) were not fully available. The table highlights both the harmonized design elements and the necessary adaptations inherent to real-world data emulation.

	KEYNOTE 189	eKN189	KEYNOTE 407	eKN407	Impower 133	eIMP133	PACIFIC	ePACIFIC
Eligibility	metastatic nonsquamous NSCLC, ECOG 0-1	approximated via registry variables	metastatic squamous NSCLC, ECOG 0-1	approximated via registry variables	ES-SCLC, ECOG 0-1	approximated via registry variables	stage III NSCLC treated with concurrent chaemoradiotherapy, ECOG 0-1	approximated via registry variables
Treatment	pembrolizumab + pemetrexed/platinum vs pemetrexed/platinum	same regimens identified	pembrolizumab + carboplatin/paclitaxel(nab) vs carboplatin/paclitaxel(nab)	same regimens identified	pembrolizumab + carboplatin/etoposide vs carboplatin/etoposide	same regimens identified	with durvalumab consolidation vs without consolidation	same regimens identified
Time zero	randomisation date	treatment initiation	randomisation date	treatment initiation	randomisation date	treatment initiation	randomisation date	end date of radiotherapy
Endpoint	OS, ORR	OS, registry-derived ORR	OS, ORR	OS, registry-derived ORR	OS, ORR	OS, registry-derived ORR	OS, ORR	OS, registry-derived ORR
Key deviations	RECIST	registry approximations	RECIST	registry approximations	RECIST	registry approximations	RECIST	registry approximations
	PD-L1 completeness	exploratory subgroup analysis	median age: 65years	median age: 68years	median age: 64years	median age: 66years	PD-L1 completeness	exploratory subgroup analysis
	brain metastasis 17%	brain metastasis 33%	brain metastasis 7.7%	brain metastasis 14.8%	brain metastasis 8.5%	brain metastasis 30.0%	stage IIIB: 44.5%	stage IIIB: 63.2%
	cisplatin use 28%	cisplatin use 10.6%	Nab paclitaxel: 39.9%	nab paclitaxel: 60%				

Table S1. Target trial emulation framework and alignment of pivotal randomized controlled trials with registry-based emulations

Figures S1a-S1d present covariate balance diagnostics for each emulated trial cohort before and after propensity score-based inverse probability weighting (IPW). Love plots display standardized mean differences (SMDs) for baseline covariates between treatment groups, allowing assessment of the effectiveness of weighting procedures in reducing baseline imbalances. An absolute SMD <0.1 was considered indicative of adequate covariate balance.

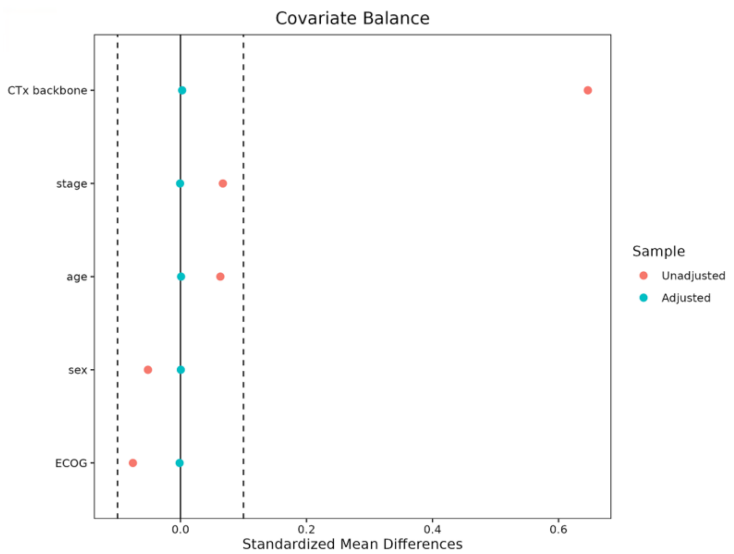

Figure S1a. Covariate balance in the eKN189 cohort. Love plot showing standardized mean differences for baseline covariates before (unadjusted) and after inverse probability weighting (adjusted) in the eKN189 cohort emulating KEYNOTE-189. Dashed vertical lines indicate the prespecified balance threshold of |0.1|.

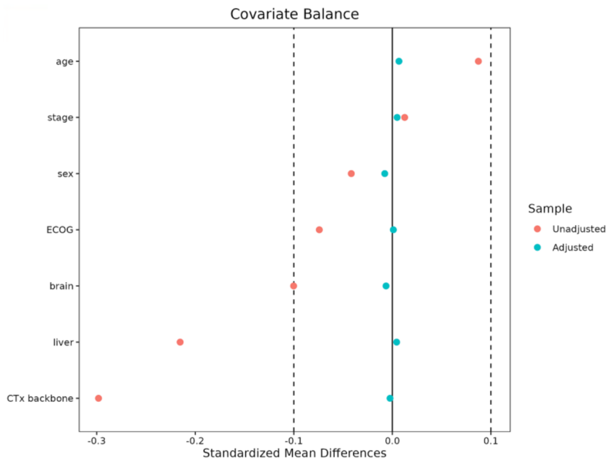

Figure S1b. Covariate balance in the eKN189 cohort. Love plot showing standardized mean differences for baseline covariates before (unadjusted) and after inverse probability weighting (adjusted) in the eKN407 cohort emulating KEYNOTE-407. Dashed vertical lines indicate the prespecified balance threshold of |0.1|.

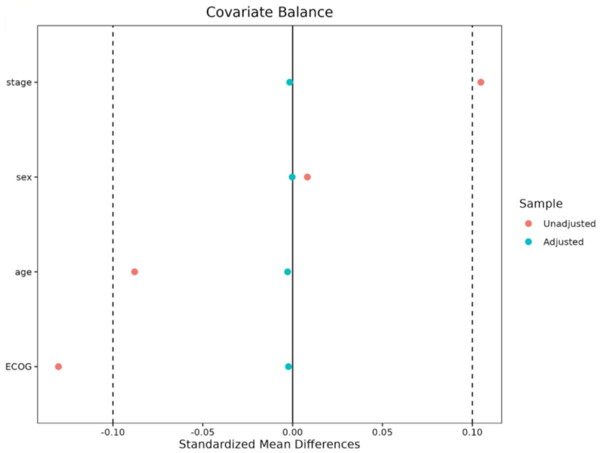

Figure S1c. Covariate balance in the eIMP133 cohort. Love plot showing standardized mean differences for baseline covariates before (unadjusted) and after inverse probability weighting (adjusted) in the eIMP133 cohort emulating IMpower133. Dashed vertical lines indicate the prespecified balance threshold of |0.1|.

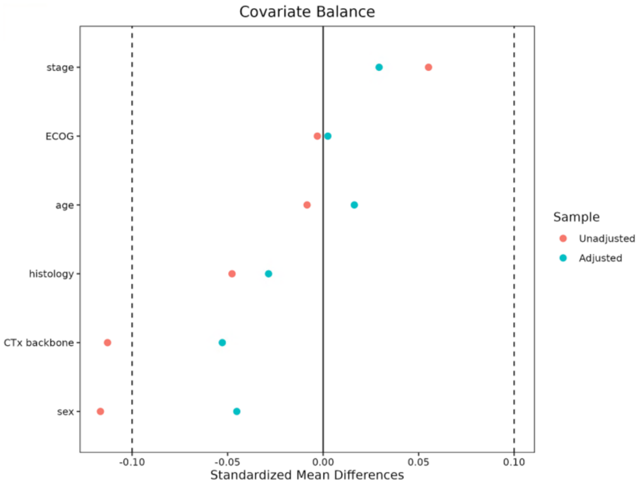

Figure S1d. Covariate balance in the ePACIFIC cohort. Love plot showing standardized mean differences for baseline covariates before (unadjusted) and after inverse probability weighting (adjusted) in the ePACIFIC cohort emulating the PACIFIC trial. Dashed vertical lines indicate the prespecified balance threshold of |0.1|
